# More secure attachment to the father and the mother is associated with fewer depressive symptoms in adolescents

**DOI:** 10.1080/03009734.2018.1439552

**Published:** 2018-03-02

**Authors:** Birgitta Kerstis, Cecilia Åslund, Karin Sonnby

**Affiliations:** a School of Health, Care and Social Welfare, Mälardalen University, Västerås, Sweden; b Centre for Clinical Research Västerås, Uppsala University, Västmanland County Hospital, Västerås, Sweden

**Keywords:** Adolescents, attachment, depression, parents

## Abstract

**Aim:**

To investigate whether more secure attachment to the father and the mother is associated with less depressive symptoms among adolescents, and to explore possible sex differences.

**Method:**

A population-based sample of adolescents completed a school-based survey assessing demographic data, attachment to father and mother, as well as depressive symptoms. Participation rate was 80% of the eligible population, and 3,988 adolescents (1,937 boys and 2,051 girls) had complete data for the analyses.

**Results:**

Paired samples *t* tests showed that participants rated their attachment to mothers as slightly more secure than their attachment to fathers (*t* = 15.94, *P* < 0.001; boys: *t* = 5.23, *P* < 0.001; girls: *t* = 16.16, *P* < 0.001). In linear regression analyses there was an association between the outcome, number of depressive symptoms, and more secure attachment to the mother for boys (*B* = −0.532; 95% confidence interval [CI] −0.656, −0.407, *P* < 0.001) and for girls (*B* = −0.623; 95% CI −0.730, −0.516, *P* < 0.001). Analogous results were found for more secure attachment to the father for boys (*B* = −0.499; 95% CI −0.608, −0.391, *P* < 0.001) and for girls (*B* = −0.494; 95% CI −0.586, −0.401, *P* < 0.001).

**Conclusions:**

Understanding the relationship between attachment to both father and mother and depressive symptoms in adolescent boys and girls is essential for further development of strategies for prevention and treatment of depression.

## Introduction

Depression affects 6% of all adolescents each year, and before reaching adulthood 14% of adolescents will have experienced depression at least once (1,2). Consequences of adolescent depression include functional impairment, negative impact on social and educational abilities, as well as increased risk of problematic on-line gaming, physical illness, and suicide (3,4). If the negative consequences on psychosocial development and education were equated to the loss of income among adults, the annual societal costs in Sweden for the approximately 30,000 adolescents affected would be 4.5 billion SEK (5). The aetiology of depression includes genetic vulnerability as well as environmental influences (3). One protective environmental factor that may prevent mental illness is secure attachment to primary caregivers (6−8).

Attachment develops during early childhood, and it can differ for the child in relation to different caregivers (9,10). Two dimensions of attachment are the secure and the insecure attachment, and most individuals in a population are relatively secure on average (9,11,12). Attachment in adolescents is stable over time, which supports that there is a stable, underlying, and enduring construct, and the obtained attachment remains important for interpersonal behaviour throughout the lifespan (7,13). Nonetheless, there are also factors known to decrease the stability of attachment, such as family conflicts, divorce, and male sex (14). Attachment can be assessed in different ways, e.g. through the ‘Strange Situation’ procedure of Ainsworth, allowing classification of attachment (e.g. secure or insecure attachment, including subcategories of insecure attachment), or also through self-report of attachment (9,12). Differences between assessment methods may influence the results, where self-reports for example are known to more reliably identify individuals with insecure attachment compared to different levels of secure attachment (12).

Previous studies investigating associations between secure attachment and depressive symptoms in adolescents have primarily focused on attachment to one parent, attachment in general, or attachment to peers, or have not analysed boys and girls separately (6,15−17).

Puissant et al. found that secure attachment to both the father and mother decreased symptoms of depression among adolescents (*n* = 225) and that the quality of attachment to the father had a stronger effect than attachment to the mother (17). Attachment and its association with depressed mood in adolescents showed sex differences in a longitudinal study from infancy to the age of 13 years (*n* = 91) (13). Follow-up at age 13 indicated that insecure attachment to their mother among girls was associated with increased emotional sensitivity, and that high emotional sensitivity was associated with adolescent depressed mood (13). Conversely, boys with insecure attachment displayed decreased emotional sensitivity (13).

There is a need to determine whether and how secure attachment to both parents is associated with depressive symptoms in adolescents of both sexes. In the absence of such knowledge, the foundation for development of intervention strategies to address deficiencies will remain problematic. Thus, the aim of the present study was to determine whether there is an association between secure attachment to their fathers and mothers with depressive symptoms in a large adolescent population. Another aim was to explore possible sex differences. The hypothesis was that adolescents with higher self-rated secure attachment to their father and mother would report fewer depressive symptoms. No sex differences were expected.

## Materials and methods

This epidemiological study used a cross-sectional design and was part of the Survey of Adolescent Life in Västmanland (SALVe) 2012. The County Council of Västmanland in Sweden distributes the SALVe every two years to all students in the 9th grade (15−16 years old) and 11th grade (17−18 years old) to monitor psychosocial health among adolescents.

The SALVe questionnaire was completed during class hours, and participation was anonymous and voluntary. Excluded were students at special schools, students with insufficient knowledge of the Swedish language, and students from schools and classes whose principals chose not to participate (*n* = 1,642). The response rate among the 5,794 eligible students was 82% (*n* = 2,121) in the 9th grade and 79% (*n* = 2,533) in the 11th grade. For students who were absent during the original survey administration, their teachers retained questionnaires that were administered later. Thus, questionnaires from 129 late respondents were returned by mail. Of the participating students, 28 did not indicate their sex, 120 did not adequately complete the questionnaire, and 518 did not answer some of the questions included in the present study. These participants were excluded, resulting in a final sample of 3,988 participants (1,937 boys, 2,051 girls).

The study followed the Swedish guidelines for studies of social science and humanities in accordance with the Declaration of Helsinki. According to Swedish regulations, this type of anonymous study does not require ethical approval by the Regional Vetting Board.

### Measures

The SALVe questionnaire includes several established questionnaires as well as study-specific questions, including questions about depressive symptoms and attachment to the father and mother, respectively.

*Depressive symptoms*. We used the Depression Self-Rating Scale (DSRS) of the DSM-IV (A-criterion), for major depression, with a reported sensitivity of 96.1% and a specificity of 59.4% for major depression (18,19). In DSM-IV, major depression according to the A-criterion is defined as two weeks of either dysphoric mood or loss of interest or pleasure in most activities, accompanied by at least four other symptoms. These could include sleep disturbances, weight loss or gain/appetite disturbances, psychomotor agitation or retardation, fatigue or loss of energy, feelings of worthlessness or guilt, concentration disturbances, and thoughts of suicide. Because depression in adolescents is defined as either dysphoric or irritable mood, the DSRS was adapted for adolescents by the addition of one question about irritable mood (20). In this study, depressive symptomatology was analysed in two ways. Firstly, as a prevalence of depressive symptoms, where we created a summation index of reported symptoms according to the DSM-IV manual and where one symptom was counted as one point (0−9 points). Secondly, as depression according to DSRS DSM-IV A-criterion; if at least one of the general criteria was fulfilled and accompanied by at least four other symptoms, the individual was classified as depressed according to DSM-IV, A-criterion. The internal consistency of the questions assessing DSM-IV criterion A in this sample as measured by Cronbach’s alpha was 0.836.

*Attachment to father and mother*. Attachment to father and mother was measured by the Relationship Structures (ECR-RS) questionnaire, a self-report instrument with nine items (21,22). A higher mean score indicates a more secure attachment. The items are: (1) ‘It helps to turn to my father in times of need’; (2) ‘I don’t feel comfortable opening up to my father’; (3) ‘I often worry that my father doesn’t really care about me’; (4) ‘I usually discuss my problems and concerns with my father’; (5) ‘I prefer not to show my father how I feel deep down’; (6) ‘I’m afraid that my father may abandon me’; (7)’ I talk things over with my father’; (8) ‘I worry that my father won’t care about me as much as I care about him’; and (9) ‘I find it easy to depend on my father’. Participants also responded to analogous statements regarding their mother. Responses were given on a Likert scale, ranging from ‘strongly disagree’ (1 point) to ‘strongly agree’ (7 points). Reversal of the scoring for items 2, 3, 5, 6, and 8 was performed prior to creating a summation index with a range of 9−63 points. The summation index based on the nine items was then divided by nine in order to illustrate the mean value of each item. The internal consistency (Cronbach’s alpha) for the secure attachment items was 0.868 for fathers and 0.835 for mothers.

*Sex*. The participants were asked if they were a boy (1 point) or a girl (2 points).

*School year*. Participants were in either the 9th grade (1 point) or the 11th grade (2 points).

*Family constellation*. Participants were asked to report with whom they lived. Possible response options were: ‘mother’, ‘father’, ‘sometimes with my mother and sometimes with my father’, ‘stepmother’, ‘stepfather’, ‘one or several siblings’, ‘other adult relative’, ‘alone or with girl-/boyfriend’, ‘foster family’, ‘other adult person’, ‘other’. Only respondents who marked ‘mother’ and ‘father’ (‘one or several siblings’ was also allowed) and no other alternatives were coded as ‘parents living together’ (1 point); all other answer combinations were coded as ‘parents not living together’ (2 points).

*Ethnicity*. Participants were defined as ‘Scandinavian ethnicity’ (1 point) if both parents were born in Scandinavia. Participants who had at least one parent born outside Scandinavia were defined as ‘non-Scandinavian ethnicity’ (2 points).

*Subjective family socio-economic status*. Subjective family socio-economic status (SES) was reported on a seven-point Likert scale adapted from Goodman et al. as previously reported (23,24). Participants were asked to rank their family’s SES compared with the rest of society, on a scale from 1 (lowest status) to 7 (highest status).

### Statistical analyses

Descriptive statistics are presented using percentages (number) for categorical variables and means and standard deviations (SD) for non-categorical variables. Depressive symptoms were considered the dependent factor. Internal consistency of the index variables is reported as Cronbach’s alpha. Sex differences in demographic variables were analysed using the chi-square test and Mann−Whitney *U* test. Differences in depressive symptoms between late respondents and the total sample were analysed using the Mann−Whitney *U* test. Differences in mean secure attachment for fathers and mothers were analysed using paired samples *t* tests. General linear models investigated secure attachment to the father and to the mother separately in relation to depressive symptoms. These general linear models were adjusted for sex, school year, ethnicity, socio-economic status, and family constellation. As there were significant effects of sex in relation to depressive symptoms, further statistical analyses were conducted separately for boys and girls. Sex-separated linear regression analyses were adjusted for school year, ethnicity, socio-economic status, and family constellation.

The level chosen for statistical significance was *P* < 0.05. All analyses were carried out using IBM SPSS Statistics 22.0 (SPSS Inc., Chicago, IL, USA).

## Results

Paired samples *t* tests showed that participants rated their attachment to mothers as slightly more secure than their attachment to fathers (*t* = 15.94, *P* < 0.001; Boys: *t* = 5.23, *P* < 0.001; Girls: *t* = 16.16, *P* < 0.001) (Table 1).

**Table 1.** Descriptive characteristics of the study sample.

	Total		Boys		Girls		Sex differences	
	*n*	%	*n*	%	*n*	%	Chi-square	*P*
Family constellation								
Nuclear family	2590	64.9	1294	66.8	1296	63.2		
Other family constellation	1398	35.1	643	33.2	755	36.8	5.720	0.017
Ethnicity								
Scandinavian	3163	79.3	1514	78.2	1649	80.4		
Non-Scandinavian	825	20.7	423	21.8	402	19.6	3.040	0.081
School year								
9th grade	1777	44.6	869	44.9	908	44.3		
11th grade	2211	55.4	1068	55.1	1143	55.7	0.141	0.707
	*M*	*SD*	*M*	*SD*	*M*	*SD*	*Z*	*P*
Symptoms of depression	2.72	2.69	2.10	2.48	3.30	2.76	−14.71	<0.001
More secure attachment to the father	5.46	1.23	5.57	1.11	5.36	1.33	−3.623	<0.001
More secure attachment to the mother	5.75	1.04	5.67	0.96	5.82	1.11	−7.257	<0.001
Subjective socio-economic status	4.48	0.99	4.58	1.01	4.38	0.96	−6.063	<0.001

In separate general linear models adjusted for sex, age, ethnicity, socio-economic status, and family constellation, there was a prominent effect of more secure attachment to both the father (*F* = 505.46, *df* = 1,3987, *P* < 0.001) and the mother (*F* = 527.58, *df* = 1,3987, *P* < 0.001) in relation to depressive symptoms. Moreover, there was an association between sex and depressive symptoms (*F* = 262.62, *df* = 1,3987, *P* < 0.001), warranting the separation by sex in the further statistical analyses.

In sex-separated linear regression analyses, attachment to both the father and to the mother was included in the models. A more secure attachment to the father and a more secure attachment to the mother were both associated with fewer depressive symptoms. Further, for both boys and girls, higher subjective socio-economic status was associated with less depressive symptoms (*P* = 0.001 and *P* < 0.001, respectively*)*, whereas non-Scandinavian ethnicity was associated with increased prevalence of depressive symptoms (*P* < 0.001 and *P* = 0.001, respectively) (Table 2).

**Table 2.** Linear regression for more secure attachment to the father and the mother in relation to number of depressive symptoms.

		95% CI		
Variable	*B*	Lower	Higher	*P*
Boys^a^				
More secure attachment to the father	−0.499	−0.608	−0.391	<0.001
More secure attachment to the mother	−0.532	−0.656	−0.407	<0.001
Parents not living together	0.107	−0.112	0.326	0.338
Non-Scandinavian ethnicity	0.673	0.428	0.917	<0.001
Subjective socio-economic status	−0.167	−0.269	−0.064	0.001
School year (ref. lower)	0.076	−0.126	0.278	0.460
Girls^b^				
More secure attachment to the father	−0.494	−0.586	−0.401	<0.001
More secure attachment to the mother	−0.623	−0.730	−0.516	<0.001
Parents not living together	0.089	−0.142	0.321	0.449
Non-Scandinavian ethnicity	0.451	0.183	0.719	0.001
Subjective socio-economic status	−0.337	−0.453	−0.222	<0.001
School year (ref. lower)	0.068	−0.146	0.282	0.534

^a^Adjusted *R^2^* = 0.181.

^b^Adjusted *R^2^* = 0.220.

An analysis of the prevalence of depressive symptoms among late respondents compared with the total sample revealed no differences between the groups (*Z* = −0.032, *P* = 0.974).

## Discussion

The study aim was to determine whether there is an association between more secure attachment to the father and mother and depressive symptoms, as well as to explore sex differences. The main study result was that, for both boys and girls, more secure attachment to the father or mother were associated with fewer depressive symptoms. This finding agrees with previous studies showing that more secure attachment to parents is associated with less depressive/emotional symptoms in adolescents (6,13,15,17).

Additionally, the present study observed that higher subjective socio-economic status was associated with less depressive symptoms, which is consistent with prior studies (25). On the other hand, non-Scandinavian ethnicity was associated with increased prevalence of depressive symptoms. One explanation may be the lower socio-economic status among immigrant groups in comparison to non-immigrants, also shown in previous research (26).

Keskin and Cam also found an association between more secure attachment in general and fewer emotional symptoms in 384 children, 11−16 years old, with girls showing more emotional symptoms than boys, similar to the present study (15). Puissant et al. studied the impact of attachment to mothers and fathers among 225 non-clinical adolescents (17). Similarly to the present study, significant associations were reported between more secure attachment and fewer depressive symptoms; however, there was a stronger effect of secure attachment to the father than to the mother (17). In the present study, there were no such differences. This may be explained by differences in sample sizes between the study groups. The use of different instruments for measuring attachment and depressive symptoms may also have contributed, at least partly, to the divergent results. Among 203 adolescents, Oldfield et al. investigated the relation between attachment to one parent, peer attachment, and school connectedness on mental health outcomes (6). In line with the results of the present study, results showed that peer attachment or school connectedness did not overcome negative influences on emotional symptoms associated with an insecure parental attachment (6). Results of the present study are also coherent with those concerning girls in a longitudinal study by Murray et al., with follow-up at 13 years (13). There was an association between infant insecure attachment and heightened emotional sensitivity among girls, which in turn was associated with adolescent depressive symptoms. In contrast, among boys with insecure attachment, a decreased emotional sensitivity was found, contradictory to results of the present study, which showed fewer depressive symptoms in both sexes to be associated with more secure attachment (13). The larger sample size and assessment of attachment in relation to both parents in the present study are methodological aspects that may explain these differences. The methods for assessing attachment also differed. Murray et al. assessed attachment among participants at 18 months using Ainsworth’s Strange Situation, while the present study used a self-report questionnaire among adolescents (13). The degree to which these assessment methods may be comparable or correlated at different ages is unknown. However, research has shown that attachment in infancy continue to predict adolescent attachment classification and impacts interpersonal behaviour throughout the lifespan (13,27,28).

Concerning sex differences, the present study showed differences between the attachment mean score to the parents. Girls scored attachment to their mother as more secure (higher mean score) than did boys. On the other hand, boys scored their attachment as more secure (higher mean score) to their father than did girls. One can speculate that gender-related issues of adolescent development are more easily supported by the parent of the same gender as the adolescent, contributing to the found gender differences.

These results should be interpreted in the light of several limitations. First, the study used a cross-sectional design that precludes causal influence. Thus, we do not know whether the presence of depressive symptoms may have affected the self-rated less secure attachment, which could have been better explored using a longitudinal study design. Second, the study reached approximately 80% of the target population. Students who were absent from school and those who did not complete the questions of attachment and depressive symptoms may differ from the larger sample, possibly with regard to less secure attachment and frequency of depressive symptoms. These absent students may have had higher levels of depressive symptoms. However, there were no differences in depressive symptoms between late respondents compared with the larger sample. According to Miller and Smith, non-respondents tend to be similar to late respondents regarding survey studies (29). Third, only Swedish-speaking adolescents were included, which lessens the generalizability. Fourth, the measurements were solely self-reports, which carry a risk of information bias due to false or inaccurate responding. Finally, the results might not be generalizable to other populations and age groups, although the use of a relatively large adolescent sample from the general population allows high generalizability to similar contexts.

A study strength was the relatively large, population-based sample with a high response rate due to administration of the questionnaire during regular school hours. Moreover, the study measured attachment to both fathers and mothers, whereas previous studies have often focused exclusively on attachment to the mother. The large sample size also allowed analyses of sex differences.

Depressive symptoms involve suffering at an individual level, and may also have negative consequences for the well-being of the entire family, which can lead to a vicious circle adversely impacting family health (3,5,13). In addition, depressive disorders cause large costs for society (4).

In public health management, secure attachment to parents may be valuable in developing strategies for population-level health promotion. Since infant and adolescent attachment are associated, early health-care interventions promoting secure attachment between child and parents could be one part of a longitudinal evaluation of a possible model that prevents adolescent depressive symptoms. In clinical settings, randomized trials of treatment of adolescent depressive symptoms, with or without addressing insecure attachment to parents, also would be an interesting area for future research.

Promotion of more secure attachment to the father and the mother may be an interesting focus to evaluate, both for health-care policy-makers with focus on health promotion as well as design of future clinical research, in order to assess the ability to prevent depressive symptoms in adolescents.
